# Quality of life assessment in schwannomatosis – A systematic review

**DOI:** 10.1016/j.bas.2025.104279

**Published:** 2025-05-22

**Authors:** Anna C. Lawson McLean, Steffen K. Rosahl, Aaron Lawson McLean, Pascal Fehringer, Anna Freier, Denise Löschner, Marcel A. Kamp, Christian Senft, Andreas K. Demetriades

**Affiliations:** a Department of Neurosurgery, Jena University Hospital, Germany; b Comprehensive Cancer Centre Central Germany, Jena, Germany; c Department of Neurosurgery, Helios Klinikum and Health & Medical University Erfurt, Germany; d Friedrich-Schiller University, Jena, Germany; e Department of Psychosomatic Medicine and Psychotherapy, Phillips University Marburg, Marburg, Germany; f Department of Palliative Care and Neuro-palliative Care, Immanuel Klinik Rüdersdorf, Brandenburg Medical School Theodor Fontane, Germany; g Department of Neurosurgery, Royal Infirmary of Edinburgh, Edinburgh, UK

**Keywords:** Schwannomatosis, Quality of life, Health-related quality of life, Neurofibromatosis 2

## Abstract

**Introduction:**

The term schwannomatosis refers to rare genetic disorders characterized by the development of multiple tumors within the central and/or peripheral nervous system. Recent advancements in our molecular understanding of these disorders have led to a redefined conceptual framework within the field, grouping NF2-related schwannomatosis with other forms of schwannomatosis.

**Research question:**

This systematic review aims to compile and analyse existing literature on QoL in individuals with schwannomatosis.

**Methods:**

A comprehensive search of electronic databases was conducted up until January 2025. Inclusion criteria included studies evaluating QoL in adults with schwannomatosis, using validated QoL assessment tools or patient-reported outcome measures. There were no geographical or time restrictions placed on the search. Case reports and reviews were excluded from the analysis to focus on original research contributions.

**Results:**

The initial search identified 241 articles, after removal of duplicates. Three articles were added after screening references. 40 articles were selected for final analysis. These studies utilized various assessment tools, including PHQ-9 (n = 10), WHOQOL-BREF (n = 9) and the disease-specific questionnaire NFTI-QOL (n = 15). The collective findings consistently indicated compromised QoL among individuals with schwannomatosis, particularly in domains such as physical functionality, pain perception, emotional well-being, and social interactions.

**Discussion and conclusion:**

This systematic review reveals substantial variability in QoL assessment for schwannomatosis, highlighting significant physical and psychological impacts in NF2-SWN and predominant bodily pain in non-NF2-SWN patients. We call for an international, interdisciplinary consensus on standardized QoL tools to enable clearer research comparisons, guide clinical practice, and improve patient-centered care.

## Highlights

• The term schwannomatosis summarises various tumour disposition syndromes including nf2-related schwannomatosis (formerly known as neurofibromatosis type 2)
•Patients with schwannomatosis experience a severe impact on physical and emotional quality of life
•Standardised test batteries are needed for future research, ideally in the context of a multi-national approach

## 1 Introduction

The umbrella term “schwannomatoses” groups rare hereditary tumour syndromes characterised by the development of multiple schwannomas, benign tumors arising from cranial and/or peripheral nerves. The term includes NF2-related schwannomatosis (NF2-SWN), previously known as neurofibromatosis type 2. Historically, neurofibromatosis type 2 and schwannomatosis were viewed as distinct entities with unique clinical and genetic features. Recent research revealed considerable overlap between NF2-SWN and classic non-nf2-related schwannomatosis (non-NF2-SWN), suggesting a continuum within a broader spectrum of schwannomatosis-related disorders. These new insights have prompted a reconsideration of previous classifications and given rise to the proposal, in 2022, of a novel nomenclature that better reflects the interrelated nature of the schwannomatoses and separates them from neurofibromatosis (Plotkin et al., 2022).

The updated diagnostic criteria for schwannomatosis classify each disease according to the specific gene that comprises a pathogenic variant:

• NF2-related schwannomatosis
• SMARCB1-related schwannomatosis
• LZTR1-related schwannomatosis
• 22q-related schwannomatosis
• NOS (Not Otherwise Specified) - no genetic tests have yet been performed
• NEC (Not Elsewhere Classified) - pathogenic variant could not be detected

**Fig. 1:**
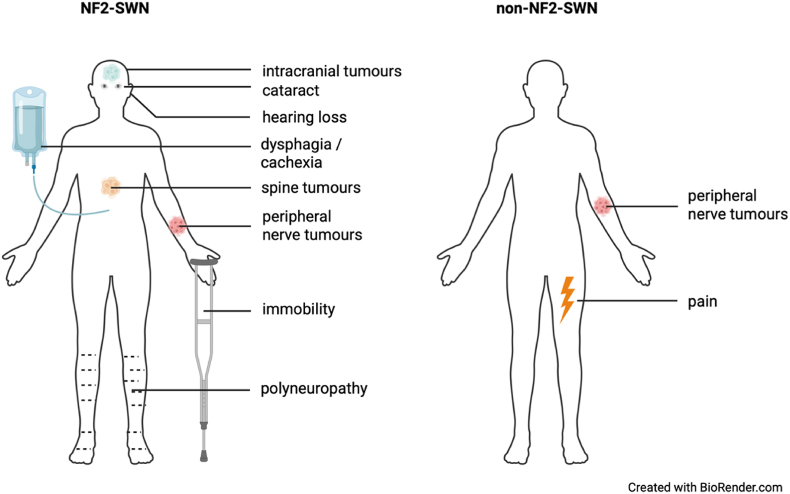
Typical clinical manifestations differ between nf2-schwannmatosis and non-NF2-schwannomatosis.

NF2-SWN and non-NF2-SWN have similarities and there can be symptomatic and phenotypical overlap between these entities. Especially patients with non-NF2-SWN and NF2-SWN mosaics can have similar symptoms. Schwannomas of the peripheral nerves are hallmarks of both disease subtypes. *Bilateral* vestibular schwannomas and meningiomas are typically found in NF2-SWN. However, people with LZTR1-related schwannomatosis can have *unilateral* vestibular schwannomas and people with SMARCB1-related schwannomatosis can develope meningiomas. Pain is a key feature of non-NF2-SWN, while NF2-SWN may present with a multitude of sensorimotor symptoms, including pain (Farschtschi et al., 2020). Studies found that 9 % of patients with a clinical diagnosis of SWN actually had NF2-SWN according to genetic analysis. Conversely, 1–2 % of patients with a clinical diagnosis of NF2-SWN actually had non-NF2-SWN according to genetic analysis (Plotkin et al., 2022; Smith et al., 2017). An overview of typical clinical manifestations of NF2-SWN and SWN ca be found in Fig. 1.

Neurofibromatosis type 1 (NF1) and NF2-related schwannomatosis (NF2-SWN) are distinct genetic disorders caused by pathogenic variants in the *nf1* and *nf2* genes, respectively. While NF1 is characterized by cutaneous signs, cognitive deficits, and neurofibromas, NF2-SWN presents with bilateral vestibular schwannomas, meningiomas, and spinal ependymomas (Farschtschi et al., 2020).

Traditionally, medical research and interventions in neurooncology have primarily focused on the physical manifestations and management of neurooncological disease. However, as the medical community recognises the holistic nature of patient well-being, there is a growing recognition that a comprehensive approach to neurooncological management (and even more so in chronic neurooncological diseases such as schwannomatosis) must encompass not only medical treatment but also the assessment and improvement of patients' quality of life (QoL). The psychosocial, emotional, and functional impact of schwannomatosis and the associated treatments cannot be underestimated, as they play a pivotal role in shaping patients' overall experiences and long-term outcomes. A 2013 systematic review that evaluated studies on QoL in patients with both NF1 and NF2-SWN by Vranceanu et al. showed that patients with these diseases had decreased QoL in comparison with the general population (Vranceanu et al., 2013). Similarly, a 2019 systematic review by Sanagoo et al. found that both NF1 and NF2-SWN had negative effects on all subscales of the SF-36 (Sanagoo et al., 2019). At the time, both reviews used the former disease classification and grouped NF1 and NF2-SWN together. The review by Vranceanu et al. included eight studies, while Sanagoo et al. included twelve. Since their publication, the disease nomenclature has been updated, and the concept of “schwannomatosis” has been redefined. Additionally, several clinical trials have since been conducted and published.

The aim of this paper is to shed light on the critical importance of evaluating and reporting the quality of life of individuals afflicted with NF2-SWN and non-NF2-SWN. Through a review of existing literature, we endeavored to highlight the need for a patient-centered approach in the management of schwannomatosis that acknowledges the significance of QoL as a primary outcome measure.

## 2 Methods

### 2.1 Literature search and selection criteria

A systematic literature search was conducted as per PRISMA guidelines to identify relevant studies exploring the quality of life in individuals with NF2-SWN and non-NF2-SWN. The search was performed in electronic databases including PubMed, Embase and ClinicalTrials.gov, utilizing the search terms "schwannomatosis," "neurofibromatosis 2," "quality of life," and "patient-reported outcomes." The search was conducted on January 17, 2025 and search criteria were not restricted by publication year, language, or study design to ensure a comprehensive collection of relevant literature.

Studies were included if they met the following criteria: (1) focused on NF2-SWN and/or SWN; (2) investigated aspects related to quality of life, including physical, psychological, social, and environmental dimensions; (3) employed validated patient-reported outcome measures (PROMs) or QoL assessment tools as primary or secondary outcomes; (4) presented original research, including observational studies, cross-sectional analyses, longitudinal studies, and intervention studies; (5) presented data on adults.

### 2.2 Data extraction and analysis

Two independent reviewers (ACLM and ALM) conducted the initial screening of titles and abstracts obtained from the literature search. Studies that met the inclusion criteria were selected for full-text review. Any discrepancies in study selection were resolved through discussion and consensus. Data were extracted in Excel by one reviewer (ACLM) and independently checked by a second reviewer (ALM). Any discrepancies were resolved through discussion.

For included studies, data were extracted using a standardised data extraction form. Extracted information included study characteristics (author, publication year, study design), participant demographics (age, gender, diagnosis), QoL assessment methods (instruments used, domains evaluated), and key findings related to QoL outcomes. If relevant information was missing in the published data corresponding authors were contacted.

Due to an anticipated heterogeneity in study designs and outcome measures, a meta-analysis was not conducted. Instead, a narrative synthesis approach was employed to summarise and qualitatively analyse the findings across studies. The synthesis focused on identifying common themes, patterns, and variations in quality-of-life outcomes amongst schwannomatosis patients.

### 2.3 Conceptualisation of quality of life

In this review, we adopted an inclusive approach to QoL, recognising it as encompassing physical, emotional, social, and functional well-being. This broad perspective aligns with the understanding that QoL is not solely limited to traditional health domains but also includes aspects that affect an individual's daily functioning and overall life satisfaction.

### 2.4 Selection of assessment tools

Given the variability in how QoL is defined and measured across studies, we included assessment tools if authors explicitly identified them as measuring QoL or patient-reported outcomes. Symptom-specific measures (e.g., hearing, tinnitus, anxiety, pain) and their effect on daily activities are part of several validated QoL assessment tools. Accordingly, we included such measures when they formed part of broader QoL assessments or were explicitly described as relevant to QoL by the original authors.

## 3 Results

### 3.1 Literature search and study selection

**Fig. 2:**
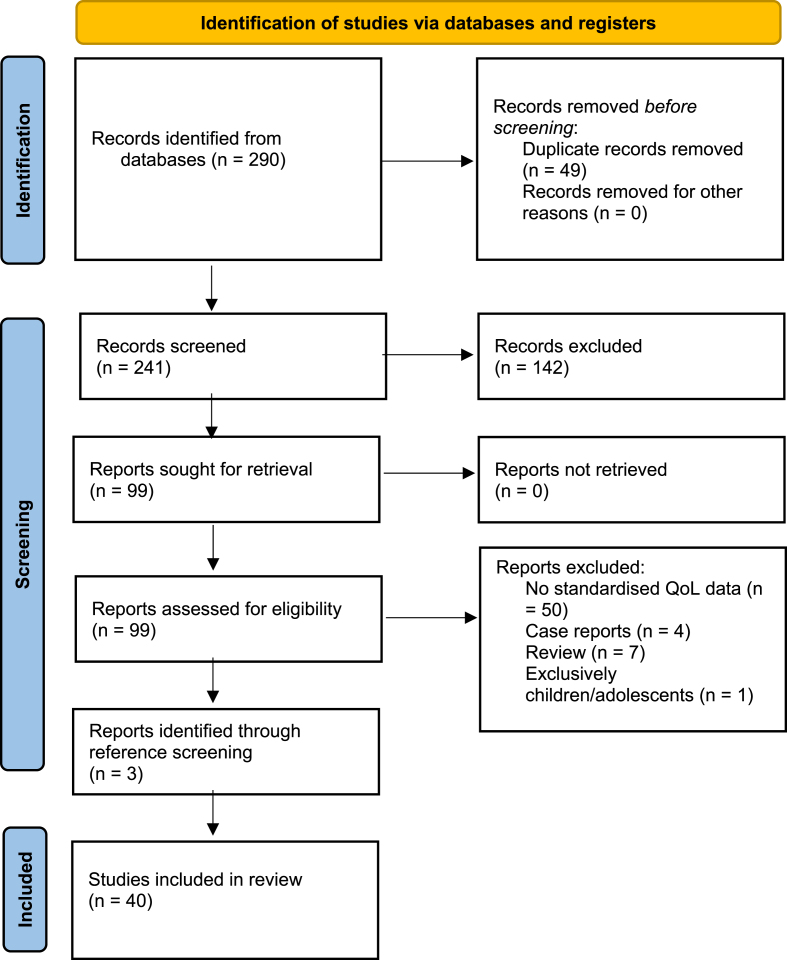
PRISMA flowchart illustrating the study selection process.

The initial literature search yielded a total of 241 relevant citations, after the removal of duplicates. Following the screening of titles and abstracts, 102 studies were selected for full-text review based on their potential relevance to the quality of life in schwannomatosis patients. After applying inclusion and exclusion criteria, a final selection of 40 studies was included in the systematic review. The Preferred Reporting Items for Systematic Reviews and Meta-Analyses (PRISMA) flowchart illustrating the study selection process is presented in Fig. 2.

### 3.2 Characteristics of included studies

The 40 included studies encompassed a variety of study designs, including cross-sectional studies (n = 20), longitudinal studies (n = 2), and clinical trials (n = 18). The studies were published between 2014 and 2024, with sample sizes ranging from 6 to 288 participants. Of these studies, 25 focused exclusively on NF2-SWN patients (see Table 1A, Table 1B).

**Table 1A:** Overview of included interventional studies.

Author and year	NCT identifier	Country	n of nf2 patients	n of classic schwannomatosis patients	Reported outcomes	Intervention
Vranceanu et al., 2014 (Vranceanu et al., 2014)		USA	6	1	CAMS-R, CIGNA Healthy Eating Survey–Section H, ESS, LOT-R, MOS, NRS, PCS, PHQ-A, PHQ-9, PHQ-15, PSS-10, PTGI, The Resiliency Scale, SLS	In-person mind–body therapy
Funes et al., 2019 (Funes et al., 2019)	NCT02811718	USA	45		CSQ-8, WHOQOL-BREF	Live-video mind-body program for patients with NF2-schwannomatosis and hearing-loss
Greenberg et al., 2019 (Greenberg et al., 2019)			45		CAMS-R, GQ-6, MOCS-A, MOS, LOT-R	
Carter et al., 2021 (Carter et al., 2021)			45		PHQ-9, PSS-10	
Vranceanu et al., 2016 (Vranceanu et al., 2016)	NCT03406208 (Pilot RCT)	USA	3	3	WHOQOL-BREF	Remote mind–body therapy
Lester 2020 (Lester et al., 2021)			3	3	WHOQOL-BREF	
Mace et al., 2021 (Mace et al., 2021)	NCT03406208	USA	32	30	CAMS-R, GAD-7, GCPS, res-6, IRI Empathy Subscale, MOCS-A, MOS, LOT-R, PHQ-9, PROMIS-PI8, PSS-10, WHOQOL-BREF	Remote mind-body therapy
Fishbein et al., 2022 (Fishbein et al., 2022)			32	20	CAMS-R, GAD-7, GCPS, GQ-6, IRI Empathy Subscale, MOCS-A, MOS, LOT-R, PHQ-9, PROMIS-PI8, PSS-10, WHOQOL-BREF	
Doorly 2022 (Doorley et al., 2021)			31	29	GAD-7, GCPS, PHQ-9, PROMIS-PI8	
Presciutti et al., 2023 (Presciutti et al., 2023)			32	30	CAMS, GAD-7, GCPS, GQ-6, IRI Empathy Subscale, MOCS-A, MOS, LOT-R, PHQ-9, PROMIS, PSS-10, WHOQOL-BREF	
Vranceanu et al., 2023 (Vranceanu et al., 2023)			32	30	CAMS, GAD-7, GCPS, GQ-6, IRI Empathy Subscale, MOCS-A, MOS, LOT-R, PHQ-9, PROMIS, PSS-10, WHOQOL-BREF	
Pietrzykowski et al., 2024 (Pietrzykowski et al., 2024)			32	30	WHOQOL-BREF	
Lester et al., 2022 (Lester et al., 2022)		USA	6	3	WHOQOL-BREF, PHQ-9, GAD-7, PROMIS	Asynchronous web-based mind-body resiliency program
Huang 2019 (Huang et al., 2018)	NCT01207687	USA	22		SSQHS, TRQ	Bevacizumab in people with NF2-schwannomatosis
Jordan et al., 2023 (Jordan et al., 2023)	NCT02831257	USA	17		NFTI-QoL, PAN-QOL	Vistusertib for progressive or symptomatic meningiomas in persons with NF2-schwannomatosis
Plotkin et al., 2019 (Plotkin et al., 2019)	NCT01767792	USA	22		NFTI-QoL, TRQ	Bevacizumab in NF2-related schwannomatosis and progressive vestibular schwannoma
Plotkin et al., 2023 (Plotkin et al., 2023)			20		NFTI-QoL, TRQ	
Plotkin et al., 2024 (Plotkin et al., 2024)	NCT04374305	USA	40		NFTI-QoL, WRS	Brigatinib in NF2-related schwannomatosis with progressive Tumors

**Table 1B:** Overview of included cross-sectional and longitudinal studies.

Author and year	Country	n of nf2 patients	n of classic schwannomatosis patients	Study design	Reported outcomes
Best et al., 2018 (Best et al., 2018)	USA	40		Cross-sectional	SSQ, VHI
Cosetti et al., 2015 (Cosetti et al., 2015)	USA	118		Cross-sectional	61-item QOL questionnaire constisting of EORTC modules QLQ-C30, BN20, H&N35, and BM22
Ferner et al., 2014 (Ferner et al., 2014)	UK	288		longitudinal	NFTI-QoL
Freier et al., 2024^44^∗	Germany	77		cross-sectional	NFTI-QOL, PHQ-9, GAD-7, SSS-8, RS-13, Loneliness Scale, OPD-SQS
Lawson McLean et al., 2023^14^∗	Germany	77		cross-sectional	NFTI-QoL, PHQ-9
Halliday et al., 2017 (Halliday et al., 2017)	UK	142		cross-sectional	NFTI-QoL
Hamoy-Jimenez et al., 2020 (Hamoy-Jimenez et al., 2020)	Canada	22		cross-sectional	EQ-5D-5L, NFTI-QoL, PROMIS-PI8, SF-36
Hornigold et al., 2012 (Hornigold et al., 2012)	UK	50		cross-sectional	EQ-5D-5L, NFTI-QoL, SF-36
Jordan et al., 2018 (Jordan et al., 2018)	USA		37	cross-sectional	SF-36, VAS
Lundin 2015 (Lundin et al., 2016)	Sweden	11		cross-sectional	NFTI-QoL
Merker et al., 2014 (Merker et al., 2014)	USA	53	50	cross-sectional	SF-36, VAS
Merker et al., 2016 (Merker et al., 2016)	USA	81		cross-sectional	SF-36
Morris et al., 2016 (Morris et al., 2016)	USA	61		longitudinal	NFTI-QoL
Mullin et al., 2022 (Mullin et al., 2022)	UK	22		cross-sectional	NFTI-QoL
Neary et al., 2010 (Neary et al., 2010)	UK	62		cross-sectional	SF-36
Quarmby et al., 2019 (Quarmby et al., 2019)	UK	177		cross-sectional	CORE-10, NFTI-QoL, WSAS
Riklin et al., 2017 (Riklin et al., 2017)	USA	33	7	cross-sectional	CAHPS-HL, FCCHL, Health LiTT, MISS
Roberts et al., 2017 (Roberts et al., 2017)	USA	112		cross-sectional	THI, Tinnitus VAS
Shukla et al., 2019 (Shukla et al., 2019)	USA	51		cross-sectional	NFTI-QOL, SF-36
Talaei-Khoei et al., 2017 (Talaei-Khoei et al., 2017)	USA	35	8	cross-sectional	PROMIS
Wolters et al., 2024 (Wolters et al., 2024)	USA	88	50	cross-sectional	Adult NF Psychosocial Survey
Yamauchi 2024 (Yamauchi and Suka, 2024)	Japan	191		cross-sectional	JMHLW Quality of life questionnaire

∗ Same cohort.

18 of the studies were part of clinical trials, 13 of which assessed psychological interventions (online and in-person mind-body therapy interventions). Five evaluated the outcomes of medication interventions (bevacizumab, brigatinib and vistusertib).

29 studies were conducted in the USA, six studies were conducted in the UK, two in Germany and one each in Sweden, Japan and Canada.

A total of 1907 patients with NF2-SWN and 189 patients with non-NF2-SWN were reported on. Considering an expected prevalence of 1:50500 for NF2-SWN and 1:126315 for non-NF2-SWN, there was an underrepresentation of non-NF2-SWN (Evans et al., 2018).

### 3.3 Quality of life assessment tools

A wide range of quality of life assessment tools were employed across the included studies. The most commonly reported instruments were the NF2 disease-specific questionnaire NFTI-QoL (reported in 15 publications referring to 14 individual studies), the mental health questionnaire PHQ-9 (reported in 10 publications referring to 5 individual studies) and the WHO quality of life questionnaire WHOQOL-BREF (reported in 9 publications referring to 5 individual studies) as well as the SF-36 (reported in 7 publications referring to 7 individual studies). Pain and its effect on QoL were most commonly assessed by PROMIS-PI8 (reported in 8 publications referring to 4 individual studies). There was no disease-specific questionnaire for non-NF2-SWN. Table 2 lists an index of all utilized questionnaires.

**Table 2:** Index of quality of life assessment tools.

Abbreviation	Meaning	Number of reporting publications	Number of reporting studies/trials
ANFPS	Adult NF Psychosocial Survey	1	1
CAHPS-HL	Consumer Assessment of Healthcare Providers and Systems Health Literacy Item Set	1	1
CAMS	Cognitive and Affective Mindfulness	2	1
CAMS-R	Cognitive and Affective Mindfulness Revised	4	1
CORE-10	Clinical Outcomes in Routine Evaluation 10	1	1
CSQ-8	Client Satisfaction Questionnaire	1	1
EORTC QLQ	European Organisation For Research And Treatment Of Cancer Quality of Life Questionnaires	1	1
EQ-5D-5L	5-level EuroQoL-5D version	2	2
FCCHL	Functional, Communicative, and Critical Health Literacy Questionnaire	1	1
GAD-7	Generalized Anxiety Disorder-7	7	3
GCPS	Graded Chronic Pain Scale	5	1
GQ-6	Gratitude Questionnaire 6-Item version	5	2
Health LiTT	Health Literacy Assessment	1	1
IRI	Interpersonal Reactivity Index	4	1
JMHLW Quality of life questionnaire	Japanese Ministry of Health, Labour and Welfare Quality of life questionnaire	1	1
LOT-R	Life Orientation Test Revised	6	3
MISS	Medical Interview Satisfaction Scale	1	1
MOCS-A	Measure of Current Status Part A	5	2
MOS	Medical Outcome Study Social Support Survey	6	3
NFTI-QoL	Neurofibromatosis 2 Impact on Quality of Life	15	14
OPD-SQS	Operationalized Psychodynamic Diagnosis-Structure Questionnaire	1	1
PAN-QOL	Penn Acoustic Neuroma Quality of Life questionnaire	1	1
PCS	Pain Catastrophizing Scale	1	1
PHQ-A	Patient-Health Questionnaire Anxiety	1	1
PHQ-9	9-item Patient Health Questionnaire	10	5
PROMIS-PI8	Pain Interference—Short Form 8a	8	4
PSS-10	Perceived Stress Scale 10-Item version	6	3
PTGI	Post Traumatic Growth Inventory	1	1
RS-13	Resilience Scale	1	1
SF-36	36-Item Short Form Survey Instrument	7	7
SLS	Satisfaction with Life Scale	1	1
SSQ	Syndney Swallowing Questionnaire	1	1
SSQS	Speech, Spatial and Qualities of Hearing Scale	1	1
SSS-8	Somatic Symptoms Scale	1	1
THI	Tinnitus Handicap Inventory	1	1
TRQ	Tinnitus Reaction Questionnaire	2	2
VAS	Visual Analogue Scale	2	2
VHI	Voice Handicap Index	1	1
WHOQOL-BREF	World Health Organization Quality of Life—Brief version	9	5
WRS	Word Recognition Score	1	1
WSAS	Work and Social Adjustment Scale	1	1

### 3.4 Quality of life domains

The SF-36 reports on various aspects of QoL and is an internationally recognised and widely used non-disease-specific questionnaire. Five independent studies from three different countries reported results of the SF-36 questionnaire exclusively in NF2-SWN patients, one reported exclusively on non-NF2-SWN patients and one reported the SF-36 results in both NF2-SWN and non-NF2-SWN patients. An overview can be found in Table 3. (The 2019 publication on NF2-SWN by Shukla et al. summarised SF-36 into different categories and did not provide a comparison with the general population and was therefore not included in Table 3. Interestingly, their publication revealed higher overall scores (better physical and emotional health) for NF2-SWN patients than the other five publications (Shukla et al., 2019). The 2018 publication by Jordan et al. on non-NF2-SWN was also not included in the table as it only used the SF-36 pain subscale. They found that bodily pain was reported to be worse than in the general population. In addition, they saw a significantly higher pain burden in non-NF2-SWN patients with *LZTR1* germline mutations than in those with *SMARCB1* mutations (Jordan et al., 2018).)

**Table 3:** Short Form-36 results compared with general populations of the respective countries. ↓ signifies worse than general population and ↔ signifies the same as general population.

SF-36 Scale	NF2-SWN					Non-NF2-SWN
	Hamoy-Jimenez et al., 2020 (Canada)	Hornigold et al., 2012 (UK)	Neary et al., 2010 (UK)	Merker et al., 2014 (USA)	Merker et al., 2016 (USA)	Merker et al., 2014 (USA)
Physical functioning	↓	↓	↓	↓	↓	↔
Physical role	↓	↓	↓	↓	↓	↓
Bodily pain	↓	↔	↓	↔	↔	↓
General health	↓	↓	↓	↓	↓	↔
Vitality	↓	↓	↓	↔	↔	↔
Social functioning	↓	↓	↓	↓	↓	↔
Emotional role	↓	↓	↓	↔	↓	↔
Mental health	↓	↓	↓	↔	↓	↔

Overall, NF2-SWN patients had statistically worse physical and emotional health than the general population of the respective countries. Interestingly, bodily pain was the only aspect of physical health where there was no statistically significant difference between NF2-SWN and general population in three out five cohorts.

The median scores of the PHQ-9 questionnaire varied from 7.0 to 9.8, which is in the range of mild depression. A recent publication found a mean of 3.2 in the general population of the UK. (Maric and Mihic, 2023) Hence, patients with schwannomatosis appear to be more prone to develop depression than the general population.

The median scores of the GAD-7 questionnaire varied from 5.0 to 8.4, which is in the range of mild anxiety. A 2017 study including 9721 subjects found a mean of 3.01 for men and 4.07 for women, respectively in the general population of Germany (Hinz et al., 2017).

The disease-specific NFTI-QoL questionnaire total score varied between studies (n = 15) with a range of 5.6–11.3 (Table 4). The mean scores of patients with sporadic vestibular schwannomas and healthy subjects were 0.47 and 0.17, respectively (Hornigold et al., 2012).

**Table 4:** Neurofibromatosis 2 Impact on Quality of Life questionnaire (NFTI-QoL) scores.

Author and year	n NF2-SWN patients	NFTI-QoL Score
Evans 2014	288	8.7
Freier et al., 2024	77	9.0^a^
Halliday et al., 2017	142	5.7–8.5^b^
Hamoy-Jimenez et al., 2020	22	9
Hornigold et al., 2012	50	9.4
Jordan et al., 2023	17	9.2
Lawson McLean et al., 2023	77	9.0^a^
Lundin 2015	11	10.3–11.3
Morris et al., 2016	61	12^c^
Mullin et al., 2022	29	9
Plotkin et al., 2023	20	7.7^d^
Plotkin et al., 2019	22	8^d^
Plotkin et al., 2024	40	8.1
Quarmby et al., 2019	104	8.9
Shukla et al., 2019	51	7.6

a Same cohort.

b Depending on genetic severity.

c At baseline.

d Both studies are part of the same randomized clinical trial.

### 3.5 Intervention studies

Among the included studies, 9 concluded clinical trials were identified, five of which assessed supportive care strategies via in-person, live-video or asynchronous web-based mind-body therapy. Vranceanu et al. reported that participants of a remote mind-body skills training had significant improvements on physical health QOL score and psychological QOL score that was sustained over twelve months after participations (Vranceanu et al., 2023). Lester et al. reported on the asynchronous web-based mind-body resiliency training that participation was associated with statistically significant positive changes in QoL, depression, anxiety, and stress (Lester et al., 2022).

Two clinical trials evaluated bevacizumab in NF2-SWN. Huang et al. reported that while there was improvement in word recognition, patients experienced no improvement of tinnitus distress (Huang et al., 2018). Plotkin et al. on the other hand reported stable QoL according to NFTI-QoL over the examination period of 98 weeks and a decrease of tinnitus distress under bevacizumab treatment (Plotkin et al., 2023). Brigatinib was reported to reduce tumour-related pain according to the NFTI-QoL item (Plotkin et al., 2024). QoL appeared to remain stable under vistusertib treatment (Jordan et al., 2023).

There are two ongoing clinical trials: NCT04163419 (“Phase 2 Study of Tanezumab in Subjects With Moderate to Severe Pain Due to Schwannomatosis”) and NCT05684692 (“Screening Trial for Pain Relief in Schwannomatosis (STARFISH)”) that both utilise the NRS for pain evaluation.

## 4 Discussion

The synthesised findings from the included studies provide a comprehensive overview of the current understanding of quality of life in schwannomatosis patients, as well as which tools and questionnaires have been used for its evaluation.

### 4.1 Quality of life assessment tools

There was a wide range of assessment tools and questionnaires that were reported. It appears that the NFTI-QoL, the PHQ-9 as well as the SF-36 are commonly used and accepted tools. A series of interventional studies and randomized controlled trials has been conducted by the US American working group utilizing the WHOQOL-BREF. This is in accordance with the REINS Patient Reported Outcome working group's recommendations.

The assessment of quality of life in schwannomatosis patients revealed several consistent domains affected by these conditions. Physical functioning was a commonly studied domain, with impairments reported in mobility, pain, and self-care. Psychological well-being was frequently explored, revealing heightened levels of anxiety, depression, and emotional distress among patients. Social functioning and role limitations due to physical and emotional health were also identified as significant concerns.

### 4.2 Comparison between NF2-SWN and non-NF2-SWN

Direct comparisons between the quality of life in NF2-SWN and non-NF2-SWN patients were limited. However, a number of studies suggested that non-NF2-SWN patients might experience milder functional impairments and fewer tumour-related symptoms compared to NF2-SWN patients. On the other hand, pain appears to be a more common symptom in non-NF2-SWN with a significant impact on these patients’ quality of life. The findings underline the importance of both extensive and specific test batteries which encompass questionnaires that address the different subtypes of schwannomatosis.

### 4.3 Geographical distortion

As our systematic review revealed, the majority of publications on quality of life in schwannomatosis patients originate from the USA. There are fewer publications from Europe and only one from Asia. This may lead to biased evaluation and decision making for patients outside of the American continent. Quality of life is not only influenced by medical care but also by surrounding factors such as social networks, financial background, healthcare systems, as well as cultural and spiritual beliefs (González et al., 2011; Li et al., 2023; OECD, 2023). There is insufficient data on how the quality of life of schwannomatosis patients differs between countries and what impact local factors may have on outcomes. Structured and comparative analysis across several countries and health care systems would be desirable. Validated translations of standardised QoL assessment tools are required. Our working group has previously translated the disease-specific NFTI-QoL into German (Lawson McLean et al., 2023). Further translations and evaluation of disease specific QoL in transnational contexts, e.g. via the European Association of Neurosurgical Societies (EANS), would be beneficial.

### 4.4 Limitations of this study

This is a retrospective analysis of the available literature and therefore carries the relevant study design limitations. The number of patients in the included studies had a relatively wide range with 6–288 patients; as the schwannomatoses are rare diseases, the overall number of patients is low. Statistical analysis is consecutively limited. In addition, the high variation of applied tests, screening tools and combinations thereof led to limited comparability.

Most of the studies in this review were published before 2022, which is before the update of the nomenclature. This means that there may be miscategorised patients in the respective groups.

In addition, it appears that there is an underrepresentation of non-NF2-SWN patients in the available literature (Evans et al., 2018). There may be several reasons, such as reporting bias or underdiagnosis of SWN. As stated above, there is an overlap of clinical manifestations of NF2-SWN and non-NF2-SWN, which may lead to misdiagnosis (Plotkin et al., 2022; Smith et al., 2017). Also, the symptom burden of non-NF2-SWN patients may be lower, and patients may not be diagnosed at all. The first reports of non-NF2-SWN date back to the late 20th century (Purcell and Dixon, 1989), while reports of NF2-SWN were already published 170 years earlier (Wishart, 1822). Therefore, there has been more established research and clinical recognition of NF2-SWN than non-NF2-SWN, which may aggravate reporting bias. The implementation of the new classification system might lead to a higher recognition of non-NF2-SWN patients as newer studies already address both NF2-SWN and non-NF2-SWN patients (NCT05684692, NCT04085159).

The heterogeneity in QoL assessment tools across studies on schwannomatosis presents a significant challenge in synthesizing data and drawing comprehensive conclusions about patient outcomes. This variation not only impedes the ability to compare results across studies but also complicates the understanding of the full impact of schwannomatosis on patients' lives. To address this issue, there is a pressing need for the standardization of QoL assessment tools in schwannomatosis research.

The Response Evaluation in Neurofibromatosis and Schwannomatosis (REiNS) International Collaboration's Patient‐Reported Outcomes (PRO) working group has systematically reviewed and published consensus guidelines for QoL endpoints in NF1, NF2, and schwannomatosis trials. Wolters et al. report that REiNS experts rated a core set of generic and NF‐specific QoL instruments and highlighted disease‐specific scales such as the NFTI-QOL scale (Wolters et al., 2021). Likewise, REiNS consensus papers have identified preferred PRO measures for key symptom domains: the Numeric Rating Scale–11 (NRS-11) and PROMIS pain/physical-function scales were recommended to capture pain intensity, pain interference and physical functioning, the Self-Assessment of Communication was endorsed for hearing function and hearing‐related QoL in NF2-SWN, and the Tinnitus Functional Index was selected as the top measure for tinnitus in NF2-SWN.

The adoption of a standardized core set of outcome measures in future studies would not only improve comparability and enable robust meta-analyses, but also increase the clinical relevance of research findings—ultimately supporting more informed patient care and targeted interventions. In the European context, implementation would be significantly strengthened by formal endorsement and strategic guidance from an international body such as the European Association of Neurosurgical Societies (EANS). A consensus statement from the EANS, grounded in data from large multinational studies, could provide a unifying framework and the necessary impetus for widespread adoption across centers.

## 5 Conclusion

This systematic review highlights the significant variability in QoL assessment methodologies for schwannomatosis patients, revealing deep impacts on physical and psychological well-being for NF2-SWN patients and a predominant effect of bodily pain in non-NF2-SWN patients. The lack of standardised QoL assessment tools complicates comparative analysis and limits our understanding of schwannomatosis's full impact. We advocate for the general implementation of an international, interdisciplinary consensus on QoL assessment in schwannomatosis research. Such standardisation would enable more patient-centered care and improved outcomes by facilitating clearer, comparative research that can guide clinical practice and improve the lives of those with schwannomatosis.

## Declaration of generative AI and AI-assisted technologies in the writing process

During the preparation of this work the authors used ChatGPT for language checking. After using this tool/service, the authors reviewed and edited the content as needed and take full responsibility for the content of the publication.

## Competing interests statement

The authors have no competing interests to declare.
